# Anti-Hypercholesterolemia Effects of Edible Seaweed Extracts and Metabolomic Changes in Hep-G2 and Caco-2 Cell Lines

**DOI:** 10.3390/life13061325

**Published:** 2023-06-05

**Authors:** Mariana Coelho, Rita Pacheco

**Affiliations:** 1 Departamento de Engenharia Química, Instituto Superior de Engenharia de Lisboa (ISEL), Rua. Conselheiro Emídio Navarro 1, 1959-007 Lisboa, Portugal; 2 Centro de Química Estrutural, Institute of Molecular Sciences, Universidade de Lisboa, 1749-016 Lisboa, Portugal

**Keywords:** cholesterol, *Eisenia bicylis*, *Porphyra tenera*, hypercholesterolemia, metabolomics, 3-hydroxy-3-methylglutaryl coenzyme A reductase, Caco-2, Hep-G2

## Abstract

Hypercholesterolemia is a major risk for the development of cardiovascular diseases (CVDs), the main cause of mortality worldwide, and it is characterized by high levels of circulating cholesterol. The drugs currently available for hypercholesterolemia control have several side effects, so it is necessary to develop new effective and safer therapies. Seaweeds serve as sources of several bioactive compounds with claimed beneficial effects. *Eisenia bicyclis* (Aramé) and *Porphyra tenera* (Nori) are edible seaweeds that were previously recognized as rich in bioactive compounds. In the present study, we aim to evaluate the anti-hypercholesterolemia effect of these two seaweed extracts and their health potential. Both extracts, but more efficiently Aramé extract, have liver 3-hydroxy-3-methylglutaryl coenzyme A reductase (HMGR) inhibitory activity as well as the capability to reduce approximately 30% of cholesterol permeation through human Caco-2 cells by simulating the intestinal lining, which is a target for hypercholesterolemia treatments. An untargeted metabolomic assay on human intestinal Caco-2 and liver Hep-G2 cell lines exposed to Aramé and Nori extracts revealed changes in the cells’ metabolism, indicating the extracts’ health beneficial effects. The metabolic pathways affected by exposure to both extracts were associated with lipid metabolism, such as phospholipids, and fatty acid metabolism, amino acid pathways, cofactors, vitamins, and cellular respiration metabolism. The effects were more profound in Aramé-treated cells, but they were also observed in Nori-exposed cells. The metabolite modifications were associated with the protection against CVDs and other diseases and to the improvement of the cells’ oxidative stress tolerance. The results obtained for the anti-hypercholesterolemia properties, in addition to the revelation of the positive impact on cell metabolism, offer an important contribution for further evaluation of these seaweed extracts as functional foods or for CVD prevention.

## 1. Introduction

According to the World Health Organization (WHO), cardiovascular diseases (CVDs) are the leading cause of mortality, both in industrialized and developing countries [1]. In 2019, around 17.9 million people died from CVDs, representing 32% of deaths world-wide. By 2030, it is estimated that at least 24 million people may die mainly from heart attack or stroke [1,2].

Hypercholesterolemia is a key factor for the development of CVDs; therefore, it is a major target in risk reduction programs [3,4]. It is characterized by high levels of low-density lipoprotein cholesterol (LDL) and very low-density lipoprotein (VLDL) or triglycerides, as well as by the decrease of high-density lipoprotein (HDL) [5,6]. Cholesterol is a lipid that plays a crucial role in many biochemical and biophysical processes; it has an irreplaceable role in human health. High levels of circulating cholesterol in the blood is a metabolic disorder or is associated with poor nutrition [7]. These high levels of cholesterol may promote atheromatous plaque deposits in the arteries, a condition known as atherosclerosis that leads to a series of complications including inflammation, high blood pressure, coronary heart disease, and other disorders leading to fatal thromboembolic events [8].

Cholesterol homeostasis in the organism is mainly maintained by three routes, endogenous cholesterol synthesis, diet or exogenous cholesterol intestinal absorption and excretion, and cholesterol hepatic excretion [9]. Cholesterol *de novo* biosynthesis occurs both in the intestine and in the liver through a pathway where the major regulatory enzyme is 3-hydroxy-3-methyglutaryl coenzyme A reductase (HMGR). Cholesterol exogenous absorption occurs via Niemann–Pick C1-Like 1 (NPC1L1) protein and other ABC transporters located in the apical membrane of enterocytes [5,10]. Human liver also expresses NPC1L1 and ABC transporters, and these are in the canalicular membrane facilitating cholesterol uptake in hepatic cells for biliary cholesterol excretion (reverse cholesterol transport) [5,10].

NPC1L1 protein is the molecular target of ezetimibe, which is often prescribed for reducing the small intestine cholesterol uptake [11]. Though life-threatening liver failure is rarely caused by this drug [12], used in monotherapy, ezetimibe has low efficacy as it reduces the circulating cholesterol by only 15–20% [13,14]. Therefore, ezetimibe is often prescribed in combination with statins for controlling hypercholesterolemia [7]. Statins are a family of compounds prescribed as HMGR enzyme inhibitors [12] with different behaviors regarding their absorption, bioavailability, excretion, binding ability to plasmatic proteins, and lipophilicity; however, most have considerable side effects [15]. Statins are well tolerated but may promote hepatotoxicity or kidney toxicity and other adverse effects such as diabetes mellitus type 2, specific effects on muscles, as well as neurologic and neurocognitive effects [16,17].

Even though the mechanisms of resistance and adverse effects to hypercholesterolemia treatment are not fully elucidated, the medical and scientific community points to the urgent need to identify individuals who are more likely to experience drug side effects and/or to find alternative therapies to the available drugs [15,18,19].

The combination of natural product intake with anti-hyperlipidemic drugs is an emerging strategy [20,21], mostly because numerous cases of cholesterol imbalance are a consequence of bad dietary habits and it is widely accepted that a healthy diet contributes to cardiovascular health [2]. Natural products are known to be rich in bioactive compounds [22], so there has been a growing interest in their use for improving health and wellness to reduce metabolic risk factors, in particular hypercholesterolemia [23].

As a still underutilized source of interesting bioactive compounds, marine organisms are emerging as possible suppliers for novel drugs [24]. In the literature, algae have been mentioned as having great potential bioactive capabilities against several human health concerns [25]. Furthermore, their prospective application as functional food ingredients, nutraceutical products, or drugs increases their economic importance.

Nevertheless, seaweeds, also known as macroalgae [26], have a long history in the southeast Asian regions, such as China, Japan, and South Korea, where they are traditionally consumed [27]. Evidence from epidemiological research suggests that regular seaweed consumption lowers the incidence of chronic diseases such as cancer, cardiovascular diseases, and related risk factors in these parts of the world [28].

Owing to a healthier diet profile, seaweed consumption is increasing in Western and European countries where they are considered to be novel functional foods, with claims of prolonging lifespan and preventing CVDs [29]. Seaweeds are also an abundant and sustainable marine resource and an alternative to animal products as they are a rich source of macro- and micronutrients [30]. The micronutrients are minerals, namely iron and magnesium, as well as lipids and water-soluble vitamins. Regarding macronutrients, seaweeds are rich in proteins and sulphate polysaccharides [31]. These nutrients are essential for human nutrition and development, and additionally some may have health impacts [32].

Seaweeds are taxonomically classified into three main groups, brown seaweeds (phylum Ochrophyta), red seaweeds (phylum Rhodophyta), and green seaweeds (phylum Chlorophyta) [29]. The colors of the seaweeds are associated with the pigments such as chlorophyll for green, phycobilin for red, and fucoxanthin for brown seaweeds [33]. *Eisenia bicyclis*, traditionally known as Aramé [34], is a perennial brown seaweed that belongs to the phylum Ochrophyta, class Phaephyceae, order Laminariales [29], and family Laminariaceae, and is distributed along the mid-Pacific coastlines of Korea and Japan [34]. Aramé seaweed is popular in Asian cuisine for soups and salads [35,36]. *Porphyra tenera*, traditionally known as Nori, is a red seaweed that belongs to the phylum Rhodophyta, class Rhodophyceae, order Bangiales, and family Bangiaceae [37], and is considered to be one of the most popular edible seaweeds since it is used in sushi and soups [38].

In this work, extracts from these seaweeds were evaluated for their potential against hypercholesterolemia, namely the capacity to inhibit HMG-CoA reductase and cholesterol permeation using Caco-2 cells as an *in vitro* model of the intestinal lining. In order to better understand their health potential, the study was complemented with a metabolomic analysis of the extract’s effects on liver Hep-G2 and Caco-2 cell lines. In particular, liver and intestinal cell line metabolites were seen to be affected due the cells’ exposure to the complex mixture of bioactive compounds in the extracts. Understanding the activity of these seaweed extracts in the biological systems of these cell lines is very important in order to explain the induced effects that reveal their bioactive mechanisms of action and how these modulated cell metabolic pathways are mainly associated with hypercholesterolemia and CVDs.

To the best of our knowledge, there are no studies that have evaluated the *Eisenia bicyclis* extract (Aramé) and *Porphyra tenera* extract (Nori) in greater detail to explain their mechanisms of action regarding their effect on hypercholesterolemia and on intracellular metabolites. 

## 2. Materials and Methods

The dried *Porphyra tenera* (Nori) seaweed biomass (Blue Dragon line of the Flavers-International Flavours Shop (B#JS2039J01)) and *Eisenia bicyclis* (Aramé) seaweed biomass (Seara brand (B# T20220405)) were purchased from a local store.

### 2.1. Chemicals

All solvents were of analytical grade unless otherwise specified and used without further purification: Roswell Park Memorial Institute (RPMI-1640) medium, Dulbecco’s Modified Eagle Medium (DMEM), trypsin, and glutamine from Biowhittaker^®^ Lonza. Fetal bovine serum (FBS) was obtained from Biowest; phosphate-buffered saline (PBS) was acquired from Corning (Corning, NY, USA). Antibiotic-Antimycotic solution 100x (10,000 U/mL penicillin, 10 mg/mL streptomycin and 25 μg amphotericin B/mL), as well as a cholesterol and HMGR assay kit were obtained from Sigma^®^Aldrich (St. Louis, MO, USA).

### 2.2. Preparation of Aramé and Nori Seaweed Extract

The dried and milled seaweed biomass was used to obtain the aqueous extracts. The procedure for obtaining a dried mass of Nori extract (50% g/g yield) through decoction in water of 20 g *Porphyra tenera* biomass/L was similar to a procedure already reported by our group [19] for obtaining the dried mass of Aramé extract through decoction of 33 g *Eisenia bicyclis* biomass/L of water (66% g/g yield). The seaweed biomass was mixed with water and the suspension was autoclaved at 121 °C for 15 min. Then, the suspension was filtered, frozen, and freeze-dried. In a recent report from the authors of [25], the aqueous extracts from these seaweeds were characterized according to their composition in total phenolic, protein, and polysaccharide content and the mixture of the compounds present was identified through liquid chromatography coupled with high resolution mass spectrometry. The identification of the bioactive compounds in these extracts showed that these contained different mixtures of phlorotannins, i.e., phloroglucinol polymers [25]. Aramé extract was seen to have a higher content in total phenols and to be a mixture of eckol and fucol derivatives, which are a type of phlorotannin derivative. As for Nori extract, it was seen to contain a higher amount of fucol type phlorotannins.

### 2.3. Cell Line Cultures

Hepatocellular carcinoma cell line (Hep-G2) and colorectal adenocarcinoma cell line (Caco-2) were cultured in DMEM and RPMI-1640 medium, respectively, supplemented with 10% (DMEM) and 20% (RPMI-1640) of inactivated FBS, 5 mL of antimycotic and 5 mL of L-glutamine at 37 °C in an atmosphere with 5% CO_2_. The medium was replaced every 48–72 h, and cells were trypsinized before reaching confluence using trypsin 1x.

### 2.4. Inhibition of Enzyme HMG-CoA Reductase (HMGR)

To determine HMGR inhibition, the procedure described in the assay kit (CS1090) was followed. In this assay, the decrease in NADPH (nicotinamide adenine dinucleotide phosphate reduced) absorbance was measured in the presence of HMG-CoA substrate. The initial rate of the enzymatic reaction was quantified by measuring the absorbance at 340 nm for 3 min (V[compound]). A control reaction was carried out using water instead of the extract solution, and this initial rate which was considered 100% of the enzymatic activity (Vcontrol). The percentage of HMGR inhibition (I) for the extracts was determined as the ratio of V[compound] and Vcontrol. All of the assays were carried out in triplicate. The concentration of the extracts used in the reaction mixture was 0.25 mg/mL.

### 2.5. In Vitro Studies in Caco-2 Cell Monolayers Simulating the Intestinal Lining

For these assays, Caco-2 cells were seeded in 12-well Transwell microplate inserts (BD FalconTM) at a density of 5 × 10^4^ cells/cm^2^, as described in [39]. The monolayers were formed and differentiated in approximately 23 days. The medium was replaced every 48 h. The monolayer formation was confirmed when it reached a trans-epithelial electrical resistance higher than 250 Ω/cm^2^, measured using a Millicell ERS-2 V Ohm (Millipore, Burlington, MA, USA).

#### 2.5.1. Cholesterol Permeation Assay

To start the assay, the Caco-2 cell monolayers were washed with Hank’s Balanced Salt Solution (HBSS). Then, the cell monolayers were incubated for 6 h with 5 mM cholesterol, and either 100 mM ezetimibe or 0.3 mg dried mass extract/mL HBSS or both were added to the samples. These assays were performed three times and compared with control cells that were only incubated with HBSS solution. For both extracts, the concentration used in contact with the cells was selected based on a previous cytotoxicity assay in Caco-2 cells [25].

Following 6 h of incubation, the solutions from both the apical side and basolateral chamber were collected and 75 μL aliquots were analyzed using RP-HPLC-DAD (VWR HITACHI, with an L-2455 detector, L-2300 column oven, L-2200 autosampler, and L-2130 pump). The separation was carried out in an isocratic mode using 50% methanol plus acetonitrile for 15 min with a flow of 1 mL/min. The percentage of cholesterol permeation was determined assuming that 100% cholesterol permeation occurred when cells were incubated for 6 h only in the presence of cholesterol.

#### 2.5.2. Seaweed Compound Permeation Assay

Caco-2 cell monolayers were incubated for 6 h with 0.3 mg dried mass extract/mL HBSS, and once the solutions from both the apical side and basolateral chamber were collected, 25 μL was further analyzed using RP-HPLC-DAD. The separation was carried out using a gradient of trifluoroacetic acid (TFA) (0.05%) and acetonitrile (ACN) for 55 min with a flow of 0.8 mL/min as follows: 0 min, 100% TFA; 30 min, 70% ACN, 30% TFA; 40 min, 20% TFA, 80% ACN; 50 min 70% ACN, 30% TFA; A; 55 min 100% TFA. The percentage of seaweed compound permeation was calculated based on the height of the most intense seaweed peak, assuming that 100% corresponded to the 0.3 mg/mL extract solution in HBSS. These assays for performed three times and compared with the control cells incubated only with HBSS solution.

### 2.6. Metabolomic Analysis of Caco-2 and Hep-G2 Cells Treated with Seaweed Extract

Hep-G2 cells were grown in T25 flasks, as previously described, to reach confluence. Caco-2 cells were also cultivated in T25 flasks, as described previously; they were allowed to reach confluence and afterwards differentiated.

Both cells were incubated with 0.3 mg seaweed extract dried mass/mL in growth medium for 24 h at 37 °C in an atmosphere with 5% CO_2_. The concentration used was selected based on previous reported cytotoxicity assays in both cell lines [25]. Control assays with cells incubated only in growth medium were additionally performed. Twenty-four hours later, the cells were washed with PBS and the cells were collected for metabolite extraction using an adaptation of a procedure described in [40].

The extracted metabolites were analyzed using liquid chromatography (LC) in an UHPLC ELUTE autosampler coupled with high resolution mass spectrometry (HRMS) in an Impact II QToF (quadruple time of flight) mass spectrometer equipped with an electrospray source (ESI) from Bruker (Bruker DaltoniK GmbH, Bremen, Germany). Extracted metabolites were injected (5 µL) in an UHPLC chromatographic column Intensity Solo 2 RP-18, 100 × 2.1 mm, 2.0 µm column (Bruker, Bremen, Germany). The column was kept at 35 °C and the samples at 10 °C. A gradient of water with 0.1% formic acid (eluent A) and acetonitrile with 0.1% formic acid (eluent B) was used at a flow rate of 0.250 mL/min as follows: 0 min—95% A; 1.5 min—95% A; 13.5 min—25% A; 18.5 min—0% A; 21.5 min—0% A; 23.5 min—95% A; 30 min—95% A. High resolution mass spectra were acquired in ESI positive and negative modes. Mass spectra were acquired in the range of 120–1000 *m*/*z* and the mass spectrometer parameters were adjusted to optimize the signal-to-noise (S/N) ratio for the ions of interest. Briefly, −3.5 kV and +4.0 kV; end plate offset, 500 V, nebulizer gas (N_2_) 2.0 bars; dry gas (N_2_), 8 Lmin^−1^; dry heater, 200 °C; collision cell energy was set to 5.0 eV. The internal calibration was performed with 250 mL H_2_O, 50 mL iPrOH, 750 µL acetic acid, 250 µL formic acid, and 0.5 mL 1N NaOH solution in HPC mode. The acquired data were processed and compared using MetaboScape^®^ 4.0 software (Bruker Daltonik GmbH, Bremen, Germany). Four replicates were made for each condition.

Metabolites from the cells incubated with the extracts were listed by their charge/mass (*m*/*z*) ratio, retention time, and abundance, either in terms of signal intensity or area. The metabolites (retention time-*m*/*z* pairs) from cells exposed to each seaweed extract were statistically compared with listed metabolites (retention time-*m*/*z* pairs) from the control cells. MetaboScape program generates a statistical analysis and the metabolites considered as having a significant variation between the control and the cells treated with the extract were those presenting a significance level at *p*-value < 0.05 and fold change below 0.5 and above 1.7.

Multivariate statistics of the metabolites, such as a principal component analysis (PCA) [41], were performed to evaluate the degree of metabolic differences between groups, i.e., control cells versus cells exposed to the extract, but also the similarity between the assayed replicates (n = 4). Another statistical analysis was applied to assess whether the detected differences in the metabolites were statistically significant between the compared groups. Volcano plots were represented as the log_2_ mean fold changes in the abundance of the metabolites between the control cells and the extract-exposed cells plotted against −log_10_ (*p*-value). The metabolites showing a *p*-value lower than 0.05 and a fold change between 0.5 and 1.7 were considered significant. Projection on latent structure (PLS) regressions were additionally applied using MetaboScape to identify similarities and differences in metabolites and/or intensities of the cellular metabolites in the control cells relative to the extract exposed cells, assigning the importance of the altered metabolites depending on the variation found. The variable importance is a measure of the contribution of each individual metabolite for the separation between groups [41]. The cell metabolites from both groups were tentatively identified using databases such as PubChem, HMDB, and Metlin. Additionally, an analysis of the metabolic pathways associated with the changes identified in the metabolites was performed using MetaboAnalyst 5.0.

### 2.7. Data Analysis

The software used for the treatment was Microsoft^®^ Excel (Microsoft Office 365) and the results were expressed as average ± standard deviation. An additional analysis of variance was carried out using a one-way ANOVA for value comparisons, and the difference between mean values were considered significant when *p*-value < 0.05.

## 3. Results

### 3.1. Seaweed Extracts’ Anti-Hypercholesterolemia Effects

#### 3.1.1. Inhibitory Effect on HMG-CoA Reductase (HMGR)

As previously mentioned, the enzyme HMGR is the key enzyme in the cholesterol *de novo* biosynthesis and is a target for the treatment of hypercholesterolemia [11]. Synthetic drugs, such as statins, are commonly prescribed, but due to their side effects, novel drugs are needed. In addition to lovastatin, which was the first drug to hit the market, there are six other statins available to date: simvastatin, pravastatin, fluvastatin, atorvastatin, rosuvastatin, and pitavastatin [42].

The potential of marine origin products for the treatment of hypercholesterolemia is still largely unexplored, though there are several that show or are claimed to have lipid lowering activities [43]. *Porphyra tenera* (Nori) and *Eisenia bicyclis* (Aramé) extracts may affect the cholesterol *de novo* biosynthesis. HMGR inhibitory activity of these extracts was evaluated and compared with pravastatin. Though there were previously reported results by our group for Aramé extract [19], to the best of our knowledge this is the first report for Nori extract. The results for 0.25 mg/mL solution of Nori dried extract, Aramé extract at the same concentration [19], and pravastatin are shown in Table 1. The Nori extract exhibits a lower inhibition capacity than the Aramé extract with 41% and 79% HMGR inhibition, respectively, and, the positive control pravastatin shows 95% enzyme inhibition. The high value of HMGR inhibition of Aramé extract, as a natural product extract, may reflect the strong potential of edible Aramé seaweed for the reduction of cholesterol levels as a functional food targeting hypercholesterolemia. As for the concentration of the Aramé extract for the achieved 50% HMGR inhibitory activity (IC_50_), a value of 0.16 mg/mL was obtained, and an IC_50_ of 0.30 mg/mL was obtained for the Nori extract.

Regarding the differences in the bioactive compounds present in these extracts, the Aramé extract has, as previously seen, a higher content in phenolic compounds and eckol type phlorotannin than the Nori extract [25]. A previous study using brown seaweeds, such as *Ecklonia stolonifera,* also reported the effect of this eckol and dieckol containing extracts as anti-hyperlipidemic agents in rats [44].

#### 3.1.2. Effects on Cholesterol Permeation In Vitro through Human Caco-2 Cells

Human epithelial colorectal adenocarcinoma cells, Caco-2, are well characterized in an intestinal *in vitro* model with morphologic resemblance to intestinal epithelia [45]. When cultured under appropriate conditions, this cell model is accepted as a surrogate for human intestinal permeability measurements by the regulatory agencies such as the FDA and EMA, and also as a screening tool for intestinal absorption, transport, and metabolism in support of drug discovery [46].

In order to compare the impact of the two seaweed extracts in the intestinal cholesterol absorption, Caco-2 cells were cultured as a monolayer and allowed to differentiate for 21 days, simulating the human intestinal epithelial membrane. The differentiated cell permeability to cholesterol was assessed in the presence of the extracts and both in the absence or presence of ezetimibe (Ezet.), the drug prescribed for reducing diet cholesterol permeation. Ezetimibe was also used alone as a positive control.

**Figure 1:**
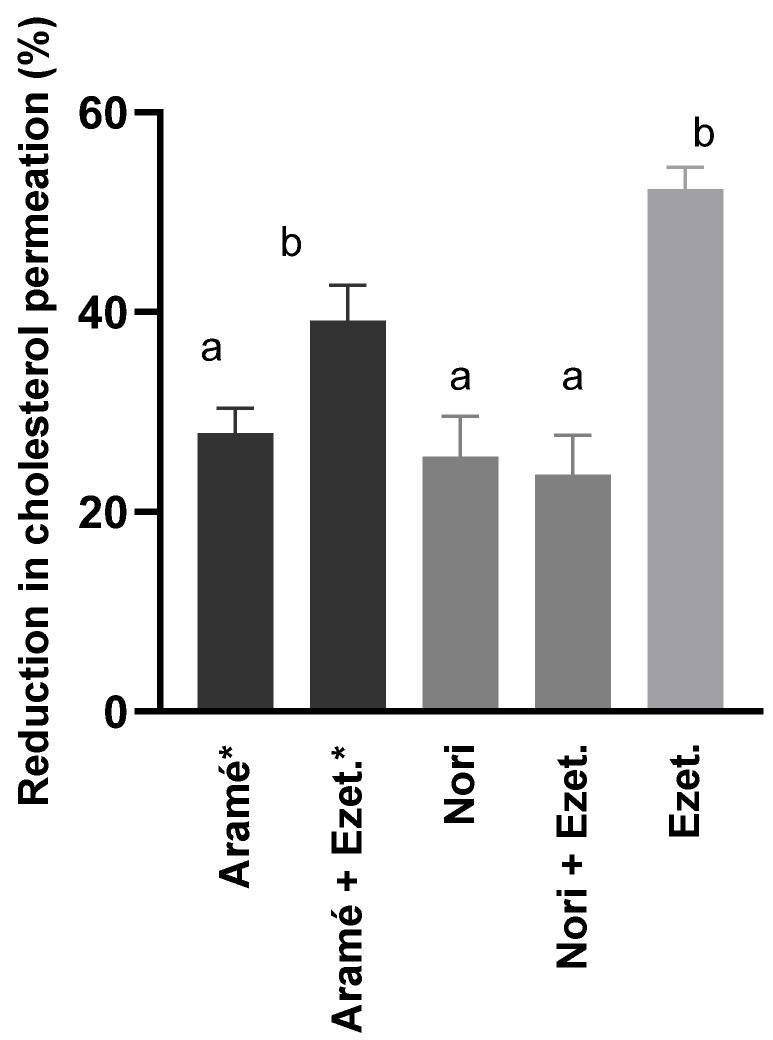
Reduction of cholesterol permeation through Caco-2 cell monolayer with extracts of Aramé and Nori. Ezetimibe (Ezet.) was used as a positive control. Caco-2 cells were differentiated for 21 days and incubated with 5 mM cholesterol for 6 h. Cholesterol was quantified using RP-HPLC-DAD, as mentioned in Section 2.5. a,b correspond to values that are statistically different between the samples under study (*p* < 0.05). * Results in [19]. Results presented correspond to the mean and standard deviation of three independent assays.

Figure 1 shows the results after 6 h of incubation with cholesterol of the percentage of reduction in cholesterol permeation at the different conditions tested. The permeability of cholesterol alone through the differentiated cells was considered 100% permeation.

It can be seen in Figure 1 that the ezetimibe (Ezet.) reduced the cholesterol permeation of the Caco-2 cells by 52%, and a similar effect was obtained when cells were in contact with ezetimibe in addition to the Aramé extract (Aramé + Ezet.). The incubation of the cells solely with any of the extracts, Aramé or Nori, only reduced cholesterol permeation by approximately 30%. Though these values are below the effect of ezetimibe, these results are considered promising as the extracts are a mixture of several compounds. Therefore, the compounds in the extracts have a promising potential for lowering cholesterol permeation in the intestinal lining, contributing to control of dietary cholesterol absorption. Considering previous results on other brown seaweeds, such as *Fucus vesiculosos* [47], and the composition of Aramé and Nori extract [25], it can be suggested that the extract compounds could interfere with the expression of cholesterol transporters in the intestinal lining NPC1L1 and ABC transporters.

This is the first report demonstrating Nori extract’s ability to inhibit cholesterol permeation in Caco-2 cells, a model of the intestinal lining. However, it can be seen in Figure 1 that in the presence of ezetimibe plus Nori (Nori + Ezet.), a lower percentage of reduction in cholesterol permeation was reached (24%) than using ezetimibe alone (52%), anticipating that the Nori extract interferes with ezetimibe’s effect. One may speculate that Nori extract’s major bioactive compounds, tentatively identified previously as smaller-sized fucol type phloroglucinol derivatives [25], may interfere with ezetimibe’s effect on the intestinal lining, which is associated with NPC1L1 protein endocytosis inhibition [48]. The awareness of interactions of seaweed compounds with drugs is limited to a few studies, mostly with anticancer drugs [49,50]; however, this is an important issue that needs to be carefully addressed considering that edible seaweeds, such as Nori, are often used in the diet.

#### 3.1.3. Seaweed Extract Permeation In Vitro through Human Caco-2 Cells

**Figure 2:**
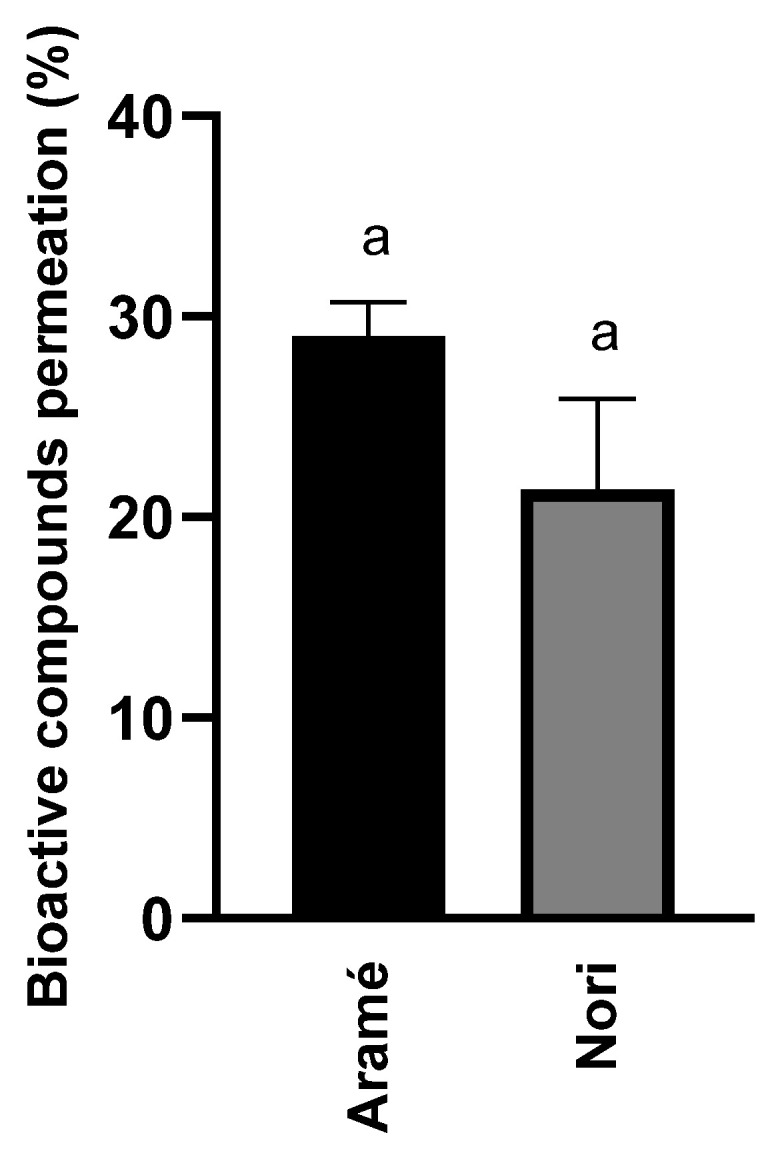
Permeation of bioactive compounds through the Caco-2 cell monolayer of Aramé and Nori extracts in the presence of cholesterol. Caco-2 cells were differentiated for 21 days and incubated with the extract for 6 h; after 6 h, the extracts were quantified using RP-HPLC-DAD, as mentioned in Section 2.5. Letter a corresponds to values that are not statistically different between the samples under study (*p* < 0.05). Results presented correspond to the mean and standard deviation of three independent assays.

A permeability assay was also conducted to evaluate whether the extract compounds permeated the gastrointestinal barrier. The results are shown in Figure 2. Overall, the results revealed that the major compounds of the analyzed seaweed extracts permeated Caco-2 cells, simulating the human intestinal epithelial membrane by less than 30%. Considering the nature of the bioactive compounds in the extracts, which are mostly lower molecular weight phlorotannin derivatives [25], it was expected that these would permeate the intestinal barrier [51,52].

These findings are significant and suggest that the extract compounds *in vivo* have the potential for permeating the gastrointestinal barrier and then moving towards other body cells, tissues, or organs, ultimately successfully reaching the liver where HMGR may be inhibited, as described in Section 3.1.1. Due to the ability of the extract compounds to permeate barriers, it seemed appropriate to further explore the impact of these seaweed extracts on the metabolism using both Caco-2 cells and human liver hepatoblastoma-derived cell line Hep-G2 cells.

#### 3.1.4. Metabolomic Effect of Seaweed Extract on Caco-2 and Hep-G2

To evaluate the mode of action and potential health effects of edible seaweed extract intake, an untargeted liquid chromatography associated with high-resolution mass spectrometry (LC-QTOF-MS) metabolomics analysis was performed. The aim of this approach was to identify key metabolites and metabolic pathways modified in liver Hep-G2 cells and intestinal Caco-2 cells in response to exposure to the extracts.

- Aramé extract

**Figure 3:**
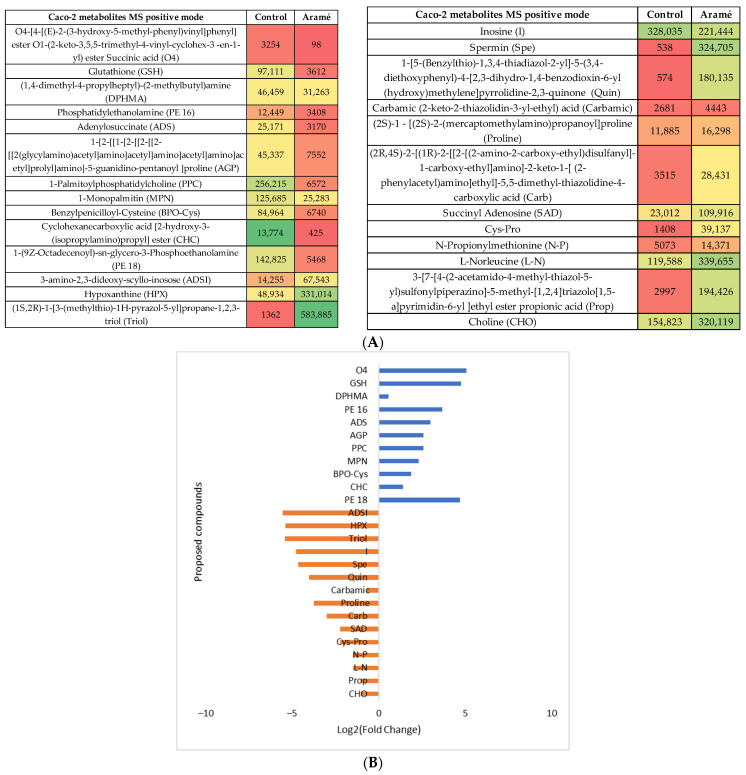
(**A**) Heatmap of intensity of the identified metabolites in Caco-2 cells in the positive mode that vary in the presence or absence of Aramé based on the mean intensities (*p* < 0.05). Control—Caco-2 cells only with culture medium. Aramé—Caco-2 cells in contact for 24 h with 0.3 mg/mL of Aramé extract. (**B**) Log_2_(Fold Change) represents the ratio in the intensity of the metabolites that are increasing in control relative to Aramé-treated cells; positive values in blue, negative values in orange.

**Figure 4:**
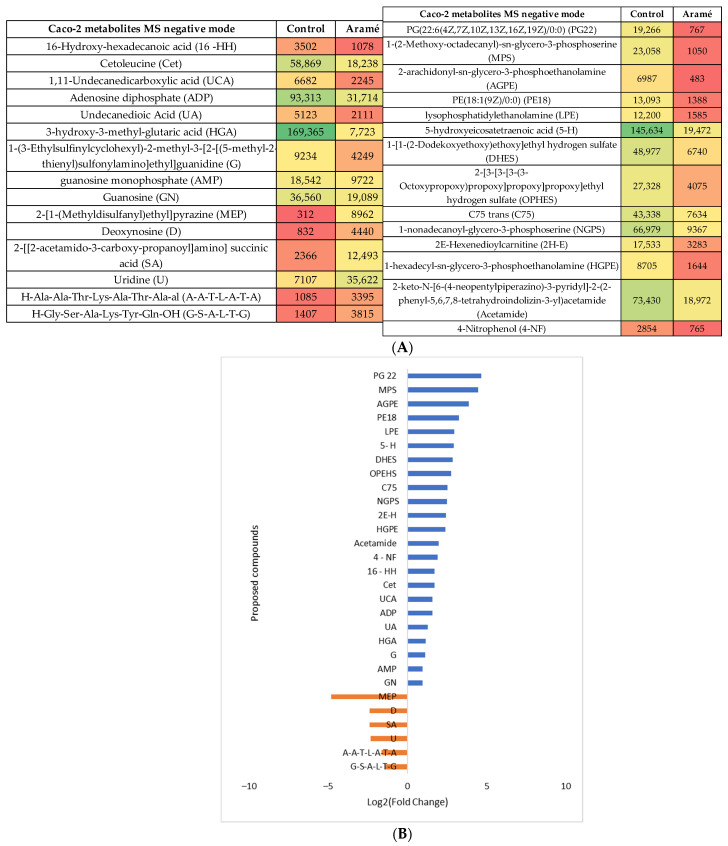
(**A**) Heatmap of the intensity of the identified metabolites in Caco-2 cells in the negative mode that vary in the presence or absence of Aramé based on the mean intensities (*p* < 0.05). Control—Caco-2 cells only with culture medium; Aramé—Caco-2 cells in contact for 24 h with 0.3 mg/mL of Aramé extract. (**B**) Log_2_(Fold Change) represents the ratio in the intensity of the metabolites that are increasing in control relative to Aramé-treated cells; positive values in blue, negative values in orange.

Caco-2 cell metabolites (Control) were compared with the metabolites from Caco-2 cells incubated in the presence of the Aramé extract. Following the statistical analysis of the list of metabolites collected through mass spectrometry, either in the MS positive or negative mode, (Supplementary Material, Figure S1) the statistically different putative metabolites were recognized and several were tentatively identified using databases such as PubChem, HMDB, and Metlin. In the heatmaps shown in Figure 3A and Figure 4A for the MS positive and negative modes, respectively, the most abundant identified metabolites are represented in green and the least abundant metabolites are shown in red. Figure 3B and Figure 4B show the relative abundance (log_2_ (fold change)) for the putative identified metabolites between the control cells (blue) and the Caco-2 cells in contact with Aramé extract (orange).

**Figure 5:**
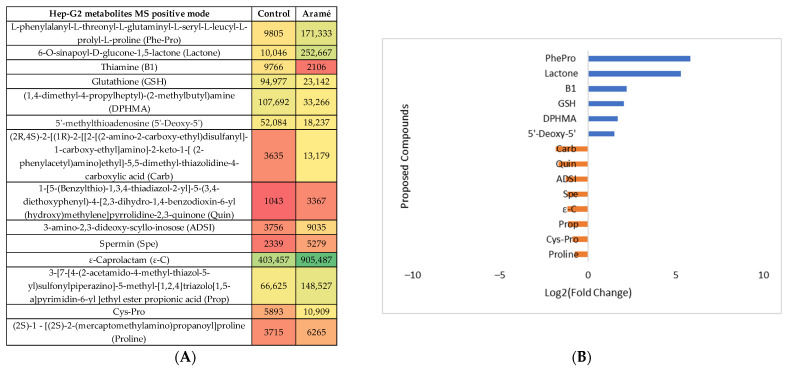
(**A**) Heatmap of the intensity of the identified metabolites in Hep-G2 cells in the positive mode that vary in the presence or absence of Aramé based on the mean intensities (*p* < 0.05). Control—Hep-G2 cells only with culture medium; Aramé—Hep-G2 cells in contact for 24 h with 0.3 mg/mL of Aramé extract. (**B**) Log_2_(Fold Change) represents the ratio in the intensity of the metabolites that are increasing in Hep-G2 control relative to Aramé-treated cells; positive values in blue, negative values in orange.

**Figure 6:**
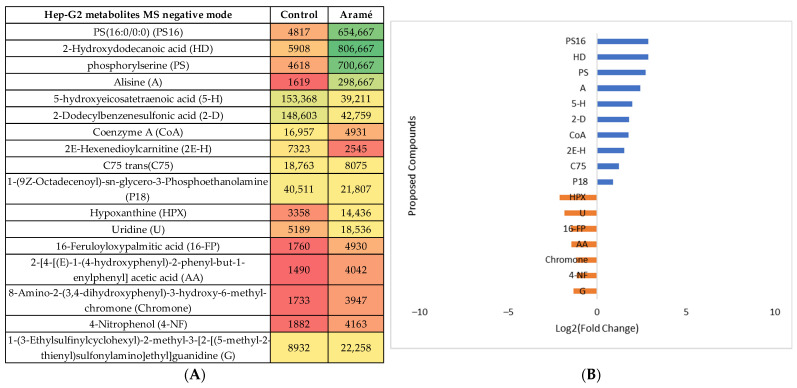
(**A**) Heatmap of the intensity of the identified metabolites in Hep-G2 cells in the negative mode that vary in the presence or absence of Aramé based on the mean intensities (*p* < 0.05). Control—Hep-G2 cells only with culture medium; Aramé—Hep-G2 cells in contact for 24 h with 0.3 mg/mL of Aramé extract. (**B**) Log_2_(Fold Change) represents the ratio in the intensity of the metabolites that are increasing in Hep-G2 control relative to Aramé-treated cells; positive values in blue, negative values in orange.

The same type of analysis and data treatment were carried out for liver Hep-G2 cells exposed to the Aramé extract. The cell-modified metabolites were extracted, MS was analyzed in both positive and negative modes, and the results were compared with the metabolites from non-exposed control cells. Following the statistical analysis (Supplementary Materials, Figure S2), the heatmaps of the statistically significantly different abundant metabolites between the control cells and Aramé-exposed cells were tentatively identified, and the differences can be seen in Figure 5A and Figure 6A for the MS positive and negative modes, respectively. Figure 5B and Figure 6B show the differences in relative abundance (log2 (fold change)) for the putative identified metabolites between Hep-G2 control cells (blue) and Hep-G2 cells in contact with Aramé extract (orange).

It was also noted through a PLS plots analysis (Figures S1 and S2c) that in both types of cells, the most important metabolites altered due to cell exposure to Aramé were mostly vitamins, phospholipids, or related fatty acid metabolites, as well as amino acids or peptides. Other types of metabolites were seen to be decreasing in exposed cells, such as reduced glutathione (GSH), which is one of the most important scavengers of reactive oxygen species (ROS) [53]. On the contrary, nucleotides, such as as uridine (U) and hypoxanthine (HPX), which is an important metabolite from the purine metabolism often associated with metabolic disorders [54], were seen to increase in the extract-treated cells.

**Figure 7:**
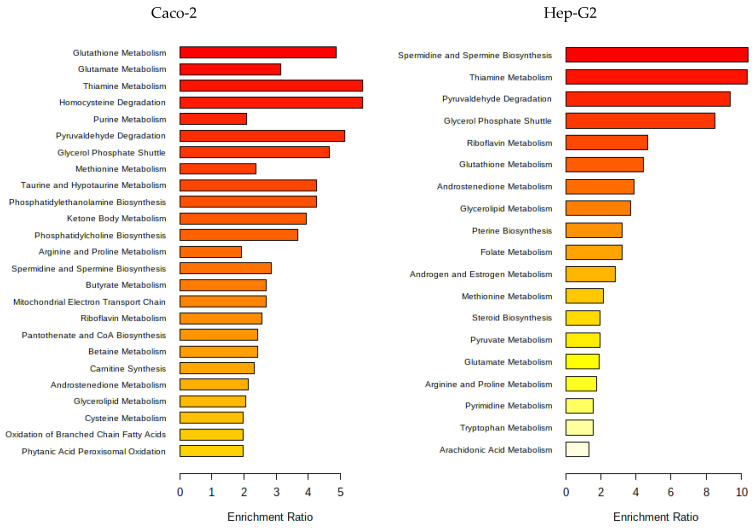
Metabolic pathways of Caco-2 and Hep-G2 cells positively affected by the presence of Aramé extract. Obtained using MetaboAnalyst 5.0 pathway enrichment analysis of significantly different metabolites between groups (*p* < 0.05). The enrichment rate is calculated as the number of hits between a particular metabolic pathway divided by the expected number of hits.

A pathway enrichment analysis of the obtained data was performed using MetaboAnalyst 5.0 tool and Figure 7 presents the metabolic pathways estimated to be affected by exposure to the Aramé extract in Caco-2 and Hep-G2 cells.

As can be seen from Figure 7, the metabolic pathways most affected in both types of cells when exposed to Aramé extract are those associated with lipid metabolism such as phospholipids and fatty acid metabolism. Furthermore, the pathways of amino acid metabolism, cofactors, and vitamins, as well as the energy metabolism of cellular respiration, namely catabolism or oxidation, were identified to be altered when the cells are exposed to the Aramé extract. 

Modifications in several of these pathways have been associated with CVDs besides hypercholesterolemia and oxidative stress. Sulfur amino acid pathways were reported as being associated with the risk of CVDs [8,55]. Sulfur amino acid pathways, such as homocysteine degradation pathway, methionine, and cysteine metabolism, are related pathways. Within the body tissues, the metabolism of methionine and cysteine determines the concentrations of several metabolites including coenzyme A, glutathione, and taurine [56,57,58,59]. In the cells treated with Aramé extract, a decrease in the level of GSH was detected relative to the control cells, which can be associated with a higher utilization rate of this metabolite inside the treated cells, a mechanism important to prevent oxidative stress. A modification in glutathione metabolism was seen in Hep-G2 cells exposed to a brown seaweed *Fucus vesiculosus* extract [60].

The metabolism of sulfur amino acids also has an impact on the synthesis of fatty acids and phospholipids [55]. These metabolites are important for cell membrane, especially for shielding the cells from oxidative stress, preventing several disorders such as CVDs, metabolic conditions, and neurodegenerative disorders [61]. It was seen that there were differences in the level and type of phospholipids and fatty acids between the control and Aramé-exposed cells. Phosphatidylcholine (PC) and phosphatidylethanolamine (PE) are major phospholipids in the mammalian membrane and their modifications are reported to modify the cell membrane behavior [62], which may restrict cell membrane fluidity, thus interfering with permeation processes and/or protecting from damage. Furthermore, PC is the major phospholipid component of lipoproteins and PE is its precursor. A reduction in PC/PE level is reported to significantly lower the levels of very low-density circulating lipoproteins (VLDLs) [63], and this reduction was observed in Aramé-treated cells.

The spermidine and spermine biosynthesis are also often associated with the ability to protect against CVDs [64], as these polyamines can act as anti-inflammatory, antioxidants, and free radical scavengers [65], and are also associated with the glutathione metabolism. In Aramé-exposed cells, a higher level of spermine was detected compared with the control cells.

Purine metabolism was also seen to be elevated in Aramé Hep-G2 and Caco-2-treated cells with accumulation of hypoxanthine (HPX), inosine (I), and deoxyinosine (D), which are products of ATP catabolism. Modifications in the purine pools in the cells are often exploited as a therapeutic target against several diseases such as cancer [66] and gout [67] and also central nervous system disorders [68].

In Hep-G2 cells exposed to Aramé extract, the thiamine metabolism was seen to be affected with a lower level of thiamine (vitamin B1) in treated cells relative to the control cells. It may be suggested that in extract-treated Hep-G2 cells, thiamine pool can be depleted due to a high metabolic rate, which increased the need of this vitamin, due to the thiamine diphosphate coenzyme role in the energy metabolism of carbohydrates, lipids, and amino acids [69]. It is known that under certain stress conditions, the intracellular thiamine pool is depleted, while increasing oxidative stress tolerance [70].

- Nori extract

**Figure 8:**
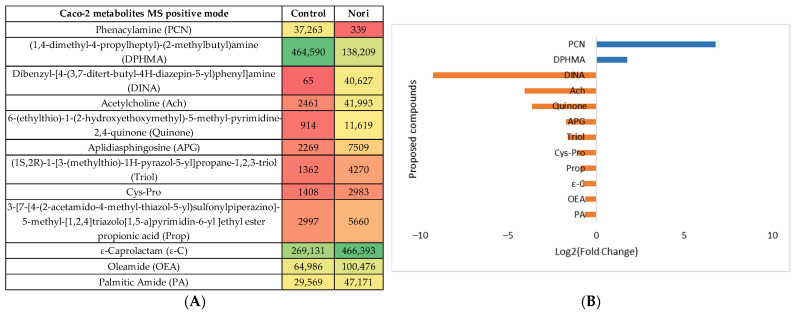
(**A**) Heatmap of the proposed compounds of Caco-2 cells that vary in the presence or absence of Nori in the positive mode based on the mean intensities (*p* < 0.05). Control—Caco-2 cells only with culture medium; Nori—Caco-2 cells in contact for 24 h with a solution of 0.3 mg/mL of Nori extract. (**B**) Log_2_(Fold Change) represents the ratio in the intensity of the metabolites that are increasing in extract-treated cells relative to the control cells’ identified metabolites; positive values in blue, negative values in orange.

**Figure 9:**
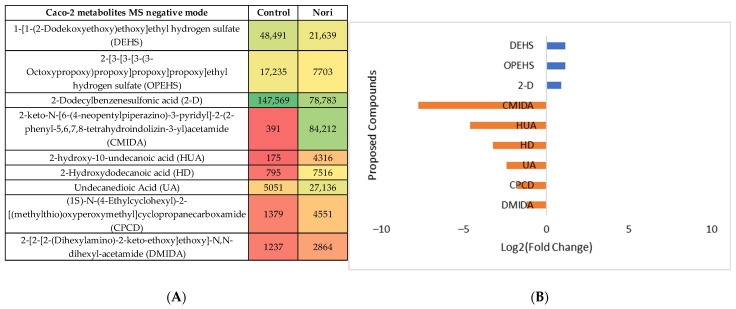
(**A**) Heatmap of the proposed compounds of Caco-2 cells that vary in the presence or absence of Nori in negative mode based on the mean intensities (*p* < 0.05). Control—Caco-2 cells only with culture medium; Nori—Caco-2 cells in contact for 24 h with a solution of 0.3 mg/mL of Nori extract. (**B**) Log_2_(Fold Change) represents the ratio in the intensity of the metabolites that are increasing in extract-treated cells relative to the control cells’ identified metabolites; positive values in blue, negative values in orange.

The same type of evaluation was performed for the metabolites from Caco-2 cells after 24 h exposure to Nori extract compared with the metabolites from non-exposed Caco-2 cells (Control). The statistical analysis is shown in Supplementary Materials Figure S3, both for the MS negative and positive modes. In the heatmaps, Figure 8A and Figure 9A show the statistically different metabolites tentatively identified using the previous mentioned databases and are colored from green to red to visualize the differences in the abundance between the control and extract-treated cells. The log_2_(Fold change) plots in Figure 8B and Figure 9B, represent the ratio between the control cell metabolite abundance and Nori-exposed cells. The most abundant metabolites in the control cells are represented by the positive blue bars and the metabolites most abundant in Nori-exposed cells are represented by orange bars.

**Figure 10:**
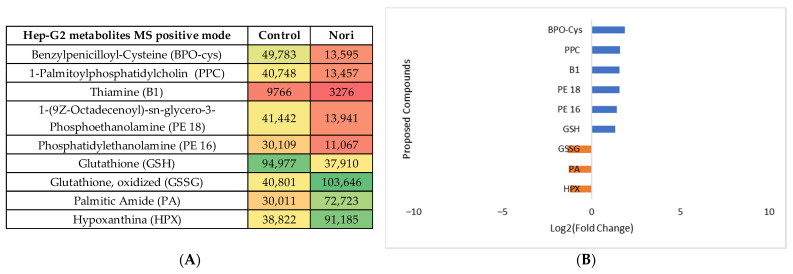
(**A**) Heatmap of the proposed compounds of Hep-G2 cells that vary in the presence or absence of Nori in positive mode based on the mean intensities (*p* < 0.05). Control—Hep-G2 cells only with culture medium; Nori—Hep-G2 cells in contact for 24 h with a solution of 0.3 mg/mL of Nori extract. (**B**) Log_2_(Fold Change) represents the ratio in the intensity of the metabolites that are increasing in extract-treated cells relative to the control cells’ identified metabolites; positive values in blue, negative values in orange.

**Figure 11:**
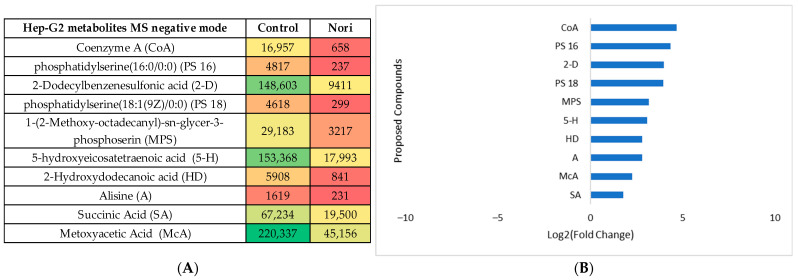
(**A**) Heatmap of the proposed compounds of Hep-G2 cells that vary in the presence or absence of Nori in negative mode based on the mean intensities (*p* < 0.05). Control—Hep-G2 cells only with culture medium; Nori—Hep-G2 cells in contact for 24 h with a solution of 0.3 mg/mL of Nori extract. (**B**) Log_2_(Fold Change) represents the ratio in the intensity of the metabolites that are increasing in extract-treated cells relative to the control cells’ identified metabolites; positive values in blue.

The same type of data analysis and evaluation were carried out for the comparison of the metabolites in Hep-G2 cells exposed to Nori extract with the metabolites from non-exposed Hep-G2 control cells. The statistical analysis is shown in Supplementary Materials Figure S4, both for the MS negative and positive modes. The heatmaps obtained after tentative identification of significantly variant metabolites are shown in Figure 10A and Figure 11A for the MS positive and negative modes, and the log_2_(Fold change) plots are shown in Figure 10B and Figure 11B.

Using the PLS plots ((Figures S3 and S4c)) and analysis of Figure 8, Figure 9, Figure 10 and Figure 11, it can be seen that overall, for both type of cells, the most important metabolites that changed between the control cells and Nori-exposed cells were mostly phospholipids or related fatty acids, nucleotides, or related purines and pyrimidines, as well as vitamins and cofactors. In the case of Hep-G2 cells exposed to Nori extract, it was additionally seen that reduced glutathione (GSH) decreased where hypoxanthine (HPX) increased, and thiamine (B1) levels decreased similarly to that previously seen for Aramé-treated Hep-G2 and Caco-2 cells. However, in the case of cells treated with Nori, the variation was less intense, as lower values of log_2_(FoldChange) were obtained relative to the cells treated with Aramé.

**Figure 12:**
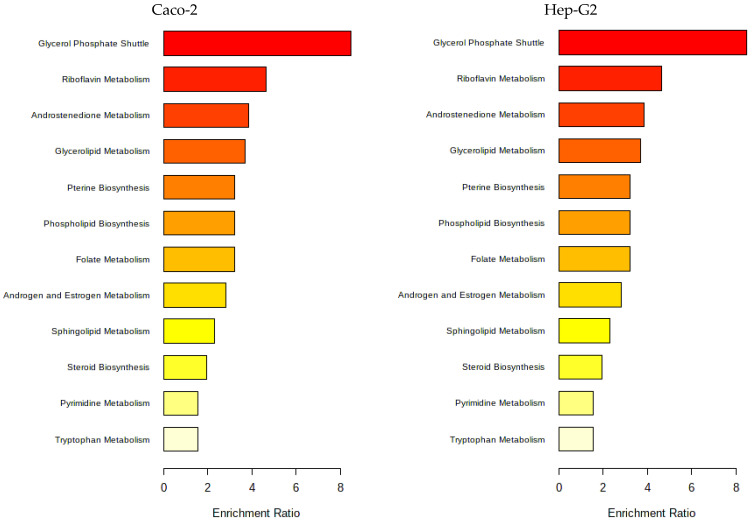
Metabolic pathways of Caco-2 and Hep-G2 cells positively affected by the proposed compounds in the presence or absence of Nori. Obtained using MetaboAnalyst 5.0 pathway enrichment analysis of significantly different metabolites between groups (*p* < 0.05). The enrichment rate is calculated as the number of hits between a particular metabolic pathway divided by the expected number of hits.

As a pathway analysis can give a more intuitive interpretation of the modifications, the metabolites were mapped to the metabolic pathways using Metaboanalyst 5.0 pathway enrichment analysis, and Figure 12 was obtained.

In Figure 12, the pathway enrichment analysis identified that the most affected metabolic pathways in both cell types due to contact with Nori extract were lipid metabolism pathways, amino acid, cofactor and vitamin, and energy metabolism pathways, as seen also for cells of exposed to the Aramé extract.

As it was mentioned for the Aramé-treated cells, variations in cell membrane lipids seen also in Nori-treated cells, are reported to be associated with modifications in cell membrane fluidity and permeation behavior [62,63], as well as on the possible effect on decreasing lipoprotein levels, which may have beneficial effects on plasma circulating cholesterol [64]. However, in the case of cells treated with Nori, the variation was less intense, as lower values of log_2_(FoldChange) were obtained relative to the cells treated with Aramé.

In Nori-treated cells, an increase relative to the non-exposed cells in amide-derived metabolites, such as 2-keto-N-[6-(4-neopentylpiperazino)-3-pyridyl]-2-(2-phenyl-5,6,7,8-tetrahydroindolizin-3-yl) (CMIDA) was also observed. Furthermore, variation occurred in the level of amine metabolites such as (1,4-dimethyl-4-propylheptyl)-(2-methylbutyl) amine (DPHMA) decreasing in Nori-treated cells, as opposed to fatty acid amides, oleamide (OEA), and palmitic amide (PA), which were increasing relative to the control. Both of these types of metabolites may be related to modifications in the metabolism of lipids, proteins, and amino acids due to exposure to the Nori extract. In the case of fatty acid amides, it was already reported that Hep-G2 metabolites were modified by a *Fucus vesiculosus* extract [60].

Furthermore, in the case of the amino acid tryptophan metabolism, in Caco-2 cells treated with Nori extract, an increase in the levels of quinone-derived metabolites often associated with this pathway was observed; again, this was also seen in Hep-G2 and Caco-2 cells treated with Aramé, but more significantly. The formation of these types of metabolites after cell exposure to the extract may be associated with cell protection, as the quinone moiety is highly reactive, thus prescribing its use against several diseases [71].

## 4. Discussion

The potential of marine origin products, including seaweeds, for prevention or treatment of diseases is still largely unexplored. As some seaweeds and derived products are already available as nutraceuticals or claimed as functional foods, it is urgent to demonstrate and further clarify their effects on consumers’ health.

The results here reported have demonstrated that the often-consumed brown seaweed Aramé and red seaweed Nori extracts may have an effective potential against hypercholesterolemia, one of the main factors associated with the risk of CVDs. Both extracts have demonstrated *in vitro* the capacity to reduce the cholesterol *de novo* synthesis by inhibiting the HMGR enzyme, a target for hypercholesterolemia treatment. Aramé extract showed a better performance than Nori extract for the same concentration. Given the differences in the composition of these extracts, Aramé extract showed higher levels of phenolic compounds and the eckol type phlorotannin, which are absent in the Nori extract [25]. It could be suggested that the inhibition is associated with the latter. Additionally, both extracts showed the capacity to reduce cholesterol permeation in the *in vitro* gastrointestinal barrier, also a target of the hypercholesterolemia drug ezetimibe. Nevertheless, it was demonstrated for the first time that the Nori extract may interfere with the ezetimibe effect on the intestinal lining. It was also demonstrated that the compounds of Aramé and Nori extracts could permeate the gastrointestinal barrier *in vitro*, and have the potential to move towards other body cells, tissues, or organs, and hypothetically reach the liver. These results are in accordance with our previous results that demonstrated in vivo that Aramé extract reduced cholesterol levels in Wistar rats [19].

To complement these results, an untargeted metabolomic analysis was carried out in intestinal Caco-2 and liver Hep-G2 cell lines to elucidate the mechanism of action associated with the consumption of both seaweeds. Our findings demonstrated variations occurring in Aramé and Nori extract-exposed cells, and showed that these could be associated with health benefits, particularly towards metabolic and other disorders, such as CVDs and oxidative-related diseases.

The cell metabolome in contact with the extracts showed an increase or decrease in several metabolites, and these were related to alterations in the cells’ major pathways such as lipid, amino acid, vitamin, and nucleotide pathways. Using either of the extracts, the analyzed cell lines showed a decrease in GSH relative to the control cells, possibly due to a higher utilization rate of this metabolite inside the treated cells, suggesting the enhancement of cells’ antioxidant mechanisms.

Modifications in lipids could be overall associated with changes in cell membranes, but particularly a decrease in PC and PE levels, suggesting that this could contribute to reducing the formation of circulating VLDLs by these cells. Other types of alterations in key metabolites, such as spermine, quinone derivatives, and vitamin B1, with antioxidant and anti-inflammatory potential, could be associated with protection against CVDs and other diseases, as well as with the improvement of cell oxidative stress tolerance. Both extracts have been already reported as showing antioxidant activity, with Aramé extract showing a better performance than Nori extract [25]. Herein it was also noticed, that though some modifications were seen in cells exposed to either extract, these were more robust in Aramé-treated cells than in Nori-exposed cells. Furthermore, for the Aramé extract, the obtained metabolomic changes are in agreement with recent results obtained in an in vivo study of the effect of the extract in the serum of Wistar rats, showing a decrease in blood cholesterol relative to control rats [19].

## 5. Conclusions

We have presented scientific evidence about the potential of brown seaweed Aramé and red seaweed Nori extracts for managing hypercholesterolemia. Both extracts demonstrated an efficient reduction of cholesterol *in vitro*, targeting cholesterol *de novo* biosynthesis and additionally the permeation in intestinal lining, showing a similar effect as the drugs often prescribed. In order to fully explore the potential of these seaweed extracts for hypercholesterolemia treatment, further research is necessary to understand the pharmacokinetic and pharmacodynamic properties to establish the adequate dosage for therapeutic products. However, it is noteworthy that the incorporation of Aramé seaweed in a healthy and balanced diet stands as a beneficial way to prevent hypercholesterolemia and to promote health. These applications, were likewise, herein scientifically demonstrated by our metabolomic findings *in vitro*, which showed that these seaweed extracts impact in the cells’ pathways is possibly advantageous for cell protection against several disorders, including the prevention of CVDs.

## Figures and Tables

**Table 1:** HMG-CoA reductase (HMGR) inhibitory activity of 0.25 mg/mL *Eisenia bicyclis* extract (Aramé), 0.25 mg/mL *Porphyra tenera* extract (Nori), and pravastatin. Results presented correspond to the mean and standard deviation of three independent assays.

	HMGR Inhibitory Activity (%)
Nori	41 ± 3 ^a^
*pravastatin*	95 ± 1 ^b^
Aramé [19]	79 ± 7 ^c^

Superscript letters (a–c) correspond to values that are statistically different between the samples under study (*p* < 0.05).

## Data Availability

Not applicable.

## Supplementary Materials

The following supporting information can be downloaded at: https://www.mdpi.com/article/10.3390/life13061325/s1. Figure S1—Analysis of the compounds of Caco-2 cells that vary in the presence or absence of Aramé in positive (1) and negative (2) modes. (a) volcano plot; (b) PCA plot; (c) PLS plot. DPHMA—(1,4-dimethyl-4-propylheptyl)-(2-methylbutyl)amine; HPX—Hypoxanthine; Prop—3-[7-[4-(2-acetamido-4-methyl-thiazol-5-yl)sulfonylpiperazino]-5-methyl-[1,2,4]triazolo[1,5-a]pyrimidin-6-yl]ethyl ester propionic acid; I—Inosine; CO—Choline; AMP—Adenosine Monophosphate; L-N—L-Norleucine; MPN—1-Monopalmitin; GSH—Glutathione; SAD—Succinyl Adenosine; BPO-Cys—Benzylpenicilloyl-Cysteine; DHES—1-[1-(2-Dodekoxyethoxy)ethoxy]ethyl hydrogen sulfate; Acetamida—2-keto-N-[6-(4-neopentylpiperazino)-3-pyridyl]-2-(2-phenyl-5,6,7,8-tetrahydroindolizin-3-yl)acetamide; Cet—Cetoleucine; U—Uridine; MPS—1-(2-Metoxy-octadecanyl)-sn-glycero-3-phosphoserine, GN—Guanosine; PG 22—PG (22:6(4Z,7Z,10Z,13Z,16Z,19Z)/0:0); 2E-H—2E-Hexenedioylcarnitine; PE 18—1-(9Z-Octadecenoyl)-sn-glycero-3-Phosphoethanolamine; C75—C75 trans. Figure S2—Analysis of the compounds of Hep-G2 cells that vary in the presence or absence of Aramé in positive (1) and negative (2) modes. (a) volcano plot; (b) PCA plot; (c) PLS plot. ε-C—ε-Caprolactam; HPX—Hypoxanthine; Prop—3-[7-[4-(2-acetamido-4-methyl-thiazol-5-yl)sulfonylpiperazino]-5-methyl-[1,2,4]triazolo[1,5-a]pyrimidin-6-yl]ethyl ester propionic acid; DPHMA—(1,4-dimethyl-4-propylheptyl)-(2-methylbutyl)amine; GSH—Glutathione; I—Inosine; 5′–Deoxy–5′—5′-methylthioadenosine; 5-H—5-hydroxyeicosatetraenoic acid; 2-D—2-Dodecylbenzenesulfonic acid; PE 18—1-(9Z-Octadecenoyl)-sn-glycero-3-Phosphoethanolamine; U—Uridine; G—1-(3-Ethylsulfinylcyclohexyl)-2-methyl-3-[2-[(5-methyl-2-thienyl)sulfonylamino]ethyl]guanidine; C75—C75 trans; CoA—coenzyme A. Figure S3—Analysis of the compounds of Caco-2 cells that vary in the presence or absence of Nori in positive (1) and negative (2) modes. (a) volcano plot; (b) PCA plot; (c) PLS plot. DPHMA—(1,4-dimethyl-4-propylheptyl)-(2-methylbutyl) amine; ε-C—ε- Caprolactam; Ach—Acetylcholine; PCN—Phenacylamine; OEA—Oleamide; DINA—Dibenzyl-[4-(3,7-ditert-butyl-4H-diazepin-5-yl)phenyl]amine; CMIDA—2-keto-N-[6-(4-neopentylpiperazino)-3-pyridyl]-2-(2-phenyl-5,6,7,8-tetrahydroindolizin-3-yl)acetamide; 2-D—2-Dodecylbenzenesulfonic acid; DEHS—1-[1-(2-Dodekoxyethoxy)ethoxy]ethyl hydrogen sulfate; UA—Undecanedioic Acid; OPEHS—2-[3-[3-[3-(3-Octoxypropoxy)propoxy]propoxy]propoxy]ethyl hydrogen sulfate; HUA—2-hydroxy-10-undecanoic acid. Figure S4—Analysis of the compounds of Hep-G2 cells that vary in the presence or absence of Nori in positive (1) and negative (2) modes. (a) volcano plot; (b) PCA plot; (c) PLS plot. AD—Adenosine; L-N—L-Norleucine; GSSH—Glutathione; DPHMA—(1,4-dimethyl-4-propylheptyl)-(2-methylbutyl) amine; GSH—Glutathione; PA—Palmitic Amide; HPX—Hypoxanthine; BPO-Cys—Benzylpenicilloyl-Cysteine; PE18—1-(9Z-Octadecenoyl)-sn-glycero-3-Phosphoethanolamine; PPC—1-Palmitoylphosphatidylcholin; 2-D—2-Dodecylbenzenesulfonic acid; 5-H—5-hydroxyeicosatetraenoic acid; McA—Methoxyacetic Acid; SA—2-[[2-acetamido-3-carboxy-propanoyl]amino] succinic acid.

## References

[1] Amante C., Esposito T., Luccheo G., Luccheo L., Russo P., Del Gaudio P. (2022). Recapsoma®: A Novel Mixture Based on Bergamot, Ipomoea Batatas, Policosanol Extracts and Liposomal Berberine for the Treatment of Hypercholesterolemia. Life.

[2] World Health Organization Cardiovascular Diseases. https://www.who.int/cardiovascular_diseases/about_cvd/en/.

[3] Martinez-Hervas S., Ascaso J.F., Huhtaniemi I., Martini L. (2019). Hypercholesterolemia. Encyclopedia of Endocrine Diseases.

[4] Civeira F., Arca M., Cenarro A., Hegele R.A. (2022). A mechanism-based operational definition and classification of hypercholesterolemia. J. Clin. Lipidol..

[5] André R., Pacheco R., Bourbon M., Serralheiro M.L. (2021). Brown Algae Potential as a Functional Food against Hypercholesterolemia: Review. Foods.

[6] Nordestgaard B.G., Nicholls S.J., Langsted A., Ray K.K., Tybjærg-Hansen A. (2018). Advances in lipid-lowering therapy through gene-silencing technologies. Nat. Rev. Cardiol..

[7] Qian J., Li Z., Zhang X., Chen J., Ding C., Yang P., Liu Y., Shi M., Ren X., Ge J. (2022). Efficacy and Tolerability of Ezetimibe/Atorvastatin Fixed-dose Combination Versus Atorvastatin Monotherapy in Hypercholesterolemia: A Phase III, Randomized, Active-controlled Study in Chinese Patients. Clin. Ther..

[8] Database H.M. Cholesterol. https://hmdb.ca/metabolites/HMDB0000067.

[9] Luo J., Yang H., Song B.L. (2020). Mechanisms and regulation of cholesterol homeostasis. Nat. Rev. Mol. Cell Biol..

[10] Ressaissi A., Attia N., Pacheco R., Falé P.L., Serralheiro M.L.M. (2020). Cholesterol transporter proteins in HepG2 cells can be modulated by phenolic compounds present in Opuntia ficus-indica aqueous solutions. J. Funct. Foods.

[11] Burmaoglu S., Yilmaz A.O., Taslimi P., Algul O., Kilic D., Gulcin I. (2018). Synthesis and biological evaluation of phloroglucinol derivatives possessing α-glycosidase, acetylcholinesterase, butyrylcholinesterase, carbonic anhydrase inhibitory activity. Archiv. Der Pharm..

[12] Phan B.A., Dayspring T.D., Toth P.P. (2012). Ezetimibe therapy: Mechanism of action and clinical update. Vasc. Health Risk Manag..

[13] Battaggia A., Donzelli A., Font M., Molteni D., Galvano A. (2015). Clinical efficacy and safety of Ezetimibe on major cardiovascular endpoints: Systematic review and meta-analysis of randomized controlled trials. PLoS ONE.

[14] Cardiology A.C.O. Ezetimibe: The Lower the LDL-C, the Better (Even for Total Cardiovascular Events). https://www.acc.org/latest-in-cardiology/articles/2016/03/09/06/50/ezetimibe-the-lower-the-ldlc-the-better.

[15] Ward N.C., Watts G.F., Eckel R.H. (2019). Statin Toxicity. Circ. Res..

[16] Mach F., Ray K.K., Wiklund O., Corsini A., Catapano A.L., Bruckert E., De Backer G., Hegele R.A., Hovingh G.K., Jacobson T.A. (2018). Adverse effects of statin therapy: Perception vs. the evidence—Focus on glucose homeostasis, cognitive, renal and hepatic function, haemorrhagic stroke and cataract. Eur. Heart J..

[17] Collins R., Reith C., Emberson J., Armitage J., Baigent C., Blackwell L., Blumenthal R., Danesh J., Smith G.D., DeMets D. (2016). Interpretation of the evidence for the efficacy and safety of statin therapy. Lancet.

[18] Duan Y., Gong K., Xu S., Zhang F., Meng X., Han J. (2022). Regulation of cholesterol homeostasis in health and diseases: From mechanisms to targeted therapeutics. Signal Transduct. Target. Ther..

[19] Pinto S., Gaspar M.M., Ascensão L., Faísca P., Reis C.P., Pacheco R. (2022). Nanoformulation of Seaweed *Eisenia bicyclis* in Albumin Nanoparticles Targeting Cardiovascular Diseases: In Vitro and In Vivo Evaluation. Mar. Drugs.

[20] El-Tantawy W.H., Temraz A. (2019). Natural products for controlling hyperlipidemia: Review. Arch. Physiol. Biochem..

[21] Hunter P.M., Hegele R.A. (2017). Functional foods and dietary supplements for the management of dyslipidaemia. Nat. Rev. Endocrinol..

[22] Mohd Sairazi N.S., Sirajudeen K.N.S. (2020). Natural Products and Their Bioactive Compounds: Neuroprotective Potentials against Neurodegenerative Diseases. Evid. Based Complement Alternat. Med..

[23] Santini A., Novellino E. (2017). Nutraceuticals in hypercholesterolaemia: An overview. Br. J. Pharmacol..

[24] Rigogliuso S., Campora S., Notarbartolo M., Ghersi G. (2023). Recovery of Bioactive Compounds from Marine Organisms: Focus on the Future Perspectives for Pharmacological, Biomedical and Regenerative Medicine Applications of Marine Collagen. Molecules.

[25] Coelho M., Duarte A.P., Pinto S., Botelho H.M., Reis C.P., Serralheiro M.L., Pacheco R. (2023). Edible Seaweeds Extracts: Characterization and Functional Properties for Health Conditions. Antioxidants.

[26] Gómez-Guzmán M., Rodríguez-Nogales A., Algieri F., Gálvez J. (2018). Potential Role of Seaweed Polyphenols in Cardiovascular-Associated Disorders. Mar. Drugs.

[27] Yamagata K. (2021). Prevention of cardiovascular disease through modulation of endothelial cell function by dietary seaweed intake. Phytomed. Plus.

[28] Meinita M.D., Harwanto D., Choi J.-S. (2022). Seaweed Exhibits Therapeutic Properties against Chronic Diseases: An Overview. Appl. Sci..

[29] Collins K.G., Fitzgerald G.F., Stanton C., Ross R.P. (2016). Looking Beyond the Terrestrial: The Potential of Seaweed Derived Bioactives to Treat Non-Communicable Diseases. Mar. Drugs.

[30] Leandro A., Pacheco D., Cotas J., Marques J.C., Pereira L., Gonçalves A.M.M. (2020). Seaweed’s Bioactive Candidate Compounds to Food Industry and Global Food Security. Life.

[31] Choudhary B., Chauhan O.P., Mishra A. (2021). Edible Seaweeds: A Potential Novel Source of Bioactive Metabolites and Nutraceuticals With Human Health Benefits. Front. Mar. Sci..

[32] Mahadevan K. (2015). Seaweeds: A Sustainable Food Source.

[33] Peñalver R., Lorenzo J.M., Ros G., Amarowicz R., Pateiro M., Nieto G. (2020). Seaweeds as a Functional Ingredient for a Healthy Diet. Mar. Drugs.

[34] Jung H.A., Roy A., Jung J.H., Choi J.S. (2017). Evaluation of the inhibitory effects of eckol and dieckol isolated from edible brown alga *Eisenia bicyclis* on human monoamine oxidases A and B. Arch. Pharm. Res..

[35] Kim H.J., Dasagrandhi C., Kim S.H., Kim B.G., Eom S.H., Kim Y.M. (2018). In Vitro Antibacterial Activity of Phlorotannins from Edible Brown Algae, *Eisenia bicyclis* Against Streptomycin-Resistant Listeria monocytogenes. Indian J. Microbiol..

[36] Kim K.A., Kim S.M., Kang S.W., Jeon S.I., Um B.H., Jung S.H. (2012). Edible seaweed, *Eisenia bicyclis*, protects retinal ganglion cells death caused by oxidative stress. Mar. Biotechnol..

[37] Venkatraman K.L., Mehta A. (2019). Health Benefits and Pharmacological Effects of *Porphyra* Species. Plant Foods Hum. Nutr..

[38] Ichihara T., Wanibuchi H., Taniyama T., Okai Y., Yano Y., Otani S., Imaoka S., Funae Y., Fukushima S. (1999). Inhibition of liver glutathione S-transferase placental form-positive foci development in the rat hepatocarcinogenesis by *Porphyra tenera* (Asakusa-nori). Cancer Lett..

[39] Arantes A.A. (2016). Inhibition of HMG-CoA redutase activity and cholesterol permeation through Caco-2 cells by caffeoylquinic acids from *Vernonia condensata* leaves. Rev. Bras. Farmacogn..

[40] Theodoridis G.A., Gika H.G., Want E.J., Wilson I.D. (2012). Liquid chromatography-mass spectrometry based global metabolite profiling: A review. Anal Chim. Acta.

[41] Worley B., Powers R. (2013). Multivariate Analysis in Metabolomics. Curr. Metab..

[42] Climent E., Benaiges D., Pedro-Botet J. (2021). Hydrophilic or Lipophilic Statins?. Front. Cardiovasc. Med..

[43] Zhao J., Cao Q., Xing M., Xiao H., Cheng Z., Song S., Ji A. (2020). Advances in the Study of Marine Products with Lipid-Lowering Properties. Mar. Drugs.

[44] Yoon N.Y., Kim H.R., Chung H.Y., Choi J.S. (2008). Anti-hyperlipidemic effect of an edible brown algae, *Ecklonia stolonifera*, and its constituents on poloxamer 407-induced hyperlipidemic and cholesterol-fed rats. Arch. Pharm. Res..

[45] Feng D., Ohlsson L., Duan R.D. (2010). Curcumin inhibits cholesterol uptake in Caco-2 cells by down-regulation of NPC1L1 expression. Lipids Health Dis..

[46] Larregieu C.A., Benet L.Z. (2013). Drug discovery and regulatory considerations for improving in silico and in vitro predictions that use Caco-2 as a surrogate for human intestinal permeability measurements. Aaps J..

[47] André R., Pacheco R., Alves A.C., Santos H.M., Bourbon M., Serralheiro M. (2023). The hypocholesterolemic potential of the edible algae *Fucus vesiculosus*: Proteomic and quantitative PCR analysis.

[48] Ge L., Wang J., Qi W., Miao H.H., Cao J., Qu Y.X., Li B.L., Song B.L. (2008). The cholesterol absorption inhibitor ezetimibe acts by blocking the sterol-induced internalization of NPC1L1. Cell Metab..

[49] Ramos A.A., Almeida T., Lima B., Rocha E. (2019). Cytotoxic activity of the seaweed compound fucosterol, alone and in combination with 5-fluorouracil, in colon cells using 2D and 3D culturing. J. Toxicol. Environ. Health A.

[50] Eid S.Y., Althubiti M.A., Abdallah M.E., Wink M., El-Readi M.Z. (2020). The carotenoid fucoxanthin can sensitize multidrug resistant cancer cells to doxorubicin via induction of apoptosis, inhibition of multidrug resistance proteins and metabolic enzymes. Phytomedicine.

[51] Murata N., Keitoku S., Miyake H., Tanaka R., Shibata T. (2022). Evaluation on intestinal permeability of phlorotannins using Caco-2 cell monolayers. Nat. Prod. Commun..

[52] Corona G., Ji Y., Anegboonlap P., Hotchkiss S., Gill C., Yaqoob P., Spencer J.P., Rowland I. (2016). Gastrointestinal modifications and bioavailability of brown seaweed phlorotannins and effects on inflammatory markers. Br. J. Nutr..

[53] Reed M.C., Thomas R.L., Pavisic J., James S.J., Ulrich C.M., Nijhout H.F. (2008). A mathematical model of glutathione metabolism. Theor. Biol. Med. Model..

[54] Hira H.S., Samal P., Kaur A., Kapoor S. (2014). Plasma level of hypoxanthine/xanthine as markers of oxidative stress with different stages of obstructive sleep apnea syndrome. Ann. Saudi Med..

[55] Blachier F., Andriamihaja M., Blais A. (2020). Sulfur-Containing Amino Acids and Lipid Metabolism. J. Nutr..

[56] KEGG Glutathione. https://www.genome.jp/pathway/map00480.

[57] KEGG Alanine, Aspartate and Glutamate Metabolism. https://www.genome.jp/pathway/map00250.

[58] KEGG Taurine and Hypotaurine Metabolism. https://www.genome.jp/pathway/map00430.

[59] Stipanuk M.H., Ueki I. (2011). Dealing with methionine/homocysteine sulfur: Cysteine metabolism to taurine and inorganic sulfur. J. Inherit Metab Dis..

[60] André R., Guedes R., López J., Serralheiro M.L. (2021). Untargeted metabolomic study of HepG2 cells under the effect of *Fucus vesiculosus* aqueous extract. Rapid Commun. Mass Spectrom..

[61] Rehman T., Shabbir M.A., Inam-Ur-Raheem M., Manzoor M.F., Ahmad N., Liu Z.W., Ahmad M.H., Siddeeg A., Abid M., Aadil R.M. (2020). Cysteine and homocysteine as biomarker of various diseases. Food Sci. Nutr..

[62] Li Z., Agellon L.B., Allen T.M., Umeda M., Jewell L., Mason A., Vance D.E. (2006). The ratio of phosphatidylcholine to phosphatidylethanolamine influences membrane integrity and steatohepatitis. Cell Metab..

[63] Deschamps C.L., Connors K.E., Klein M.S., Johnsen V.L., Shearer J., Vogel H.J., Devaney J.M., Gordish-Dressman H., Many G.M., Barfield W. (2015). The ACTN3 R577X Polymorphism Is Associated with Cardiometabolic Fitness in Healthy Young Adults. PLoS ONE.

[64] Qiu Y., Li L., Guo X., Liu J., Xu L., Li Y. (2022). Exogenous spermine inhibits high glucose/oxidized LDL-induced oxidative stress and macrophage pyroptosis by activating the Nrf2 pathway. Exp. Ther. Med..

[65] Yu Z., Jiao Y., Zhang J., Xu Q., Xu J., Li R., Yuan W., Guo H., Sun Z., Zheng L. (2022). Effect of Serum Spermidine on the Prognosis in Patients with Acute Myocardial Infarction: A Cohort Study. Nutrients.

[66] Liu J., Hong S., Yang J., Zhang X., Wang Y., Wang H., Peng J., Hong L. (2022). Targeting purine metabolism in ovarian cancer. J. Ovarian Res..

[67] Said H.M., Reidling J.C., Ortiz A. (2002). Cellular and molecular aspects of thiamin uptake by human liver cells: Studies with cultured HepG2 cells. Biochim. Biophys. Acta (BBA)—Biomembr..

[68] Nascimento F.P., Macedo-Júnior S.J., Lapa-Costa F.R., Cezar-dos-Santos F., Santos A.R.S. (2021). Inosine as a Tool to Understand and Treat Central Nervous System Disorders: A Neglected Actor?. Front. Neurosci..

[69] Eshak E.S., Arafa A.E. (2018). Thiamine deficiency and cardiovascular disorders. Nutr. Metab Cardiovasc. Dis..

[70] Tunc-Ozdemir M., Miller G., Song L., Kim J., Sodek A., Koussevitzky S., Misra A.N., Mittler R., Shintani D. (2009). Thiamin confers enhanced tolerance to oxidative stress in Arabidopsis. Plant Physiol..

[71] Bolton J.L., Dunlap T. (2017). Formation and Biological Targets of Quinones: Cytotoxic versus Cytoprotective Effects. Chem. Res. Toxicol..

