# Supplementary material for: Anti-Hypercholesterolemia Effects of Edible Seaweed Extracts and Metabolomic Changes in Hep-G2 and Caco-2 Cell Lines
Source: Life (Basel). 2023 Jun 5;13(6):1325. doi: 10.3390/life13061325 (PMC10305398; doi:10.3390/life13061325)
Supplement: Supplementary file 1 [file life-13-01325-s001.zip › life-2374031-supplementary.pdf]

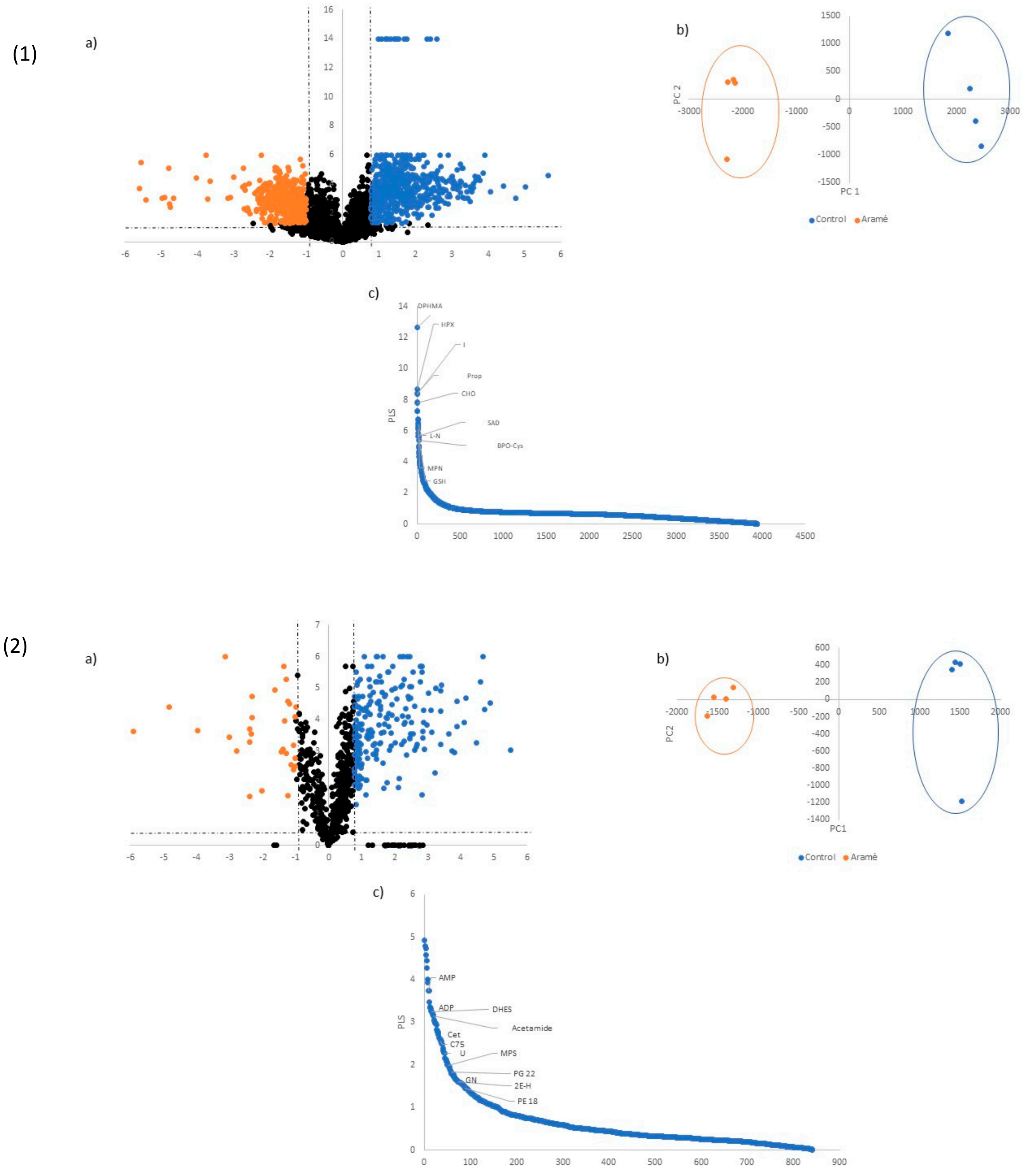

Figure S1. Analysis of compounds that vary in the presence or absence of Aramé, for Caco-2 cells, in positive (1) and negative (2) mode. (a) volcano plot; (b) PCA plot; (c) PLS plot. DPHMA—(1,4-dimethyl-4-propylheptyl)-(2-methylbutyl)amine; HPX—Hypoxanthine; Prop-3-[7-[4-(2-acetamido-4-methyl-thiazol-5-yl)sulfonylpiperazino]-5-methyl-[1,2,4]triazolo[1,5-a]pyrimidin-6-yl]ethyl ester propionic acid; I—Inosine; CO—Choline; AMP—Adenosine Monophosphate; L-N—L-Norleucine; MPN—1-Monopalmitin; GSH—Glutathione; SAD—Succinyl Adenosine; BPO-Cys—Benzylpenicilloyl-Cysteine; DHES—1-[1-(2-Dodekoxyethoxy)ethoxy]ethyl hydrogen sulfate; Acetamida—2-keto-N-[6-(4-neopentylpiperazino)-3-pyridyl]-2-(2-phenyl-5,6,7,8-tetrahydroindolizin-3-yl)acetamide; Cet—Cetoleucine; U -Uridine; MPS—1-(2-Metoxy-octadecanyl)-sn-glycero-3-phosphoserine, GN—Guanosine; PG 22—PG (22:6(4Z,7Z,10Z,13Z,16Z,19Z)/0:0); 2E-H—2E-Hexenedioylcarnitine; PE 18—1-(9Z-Octadecenoyl)-sn-glycero-3-Phosphoethanolamine; C75—C75 trans.

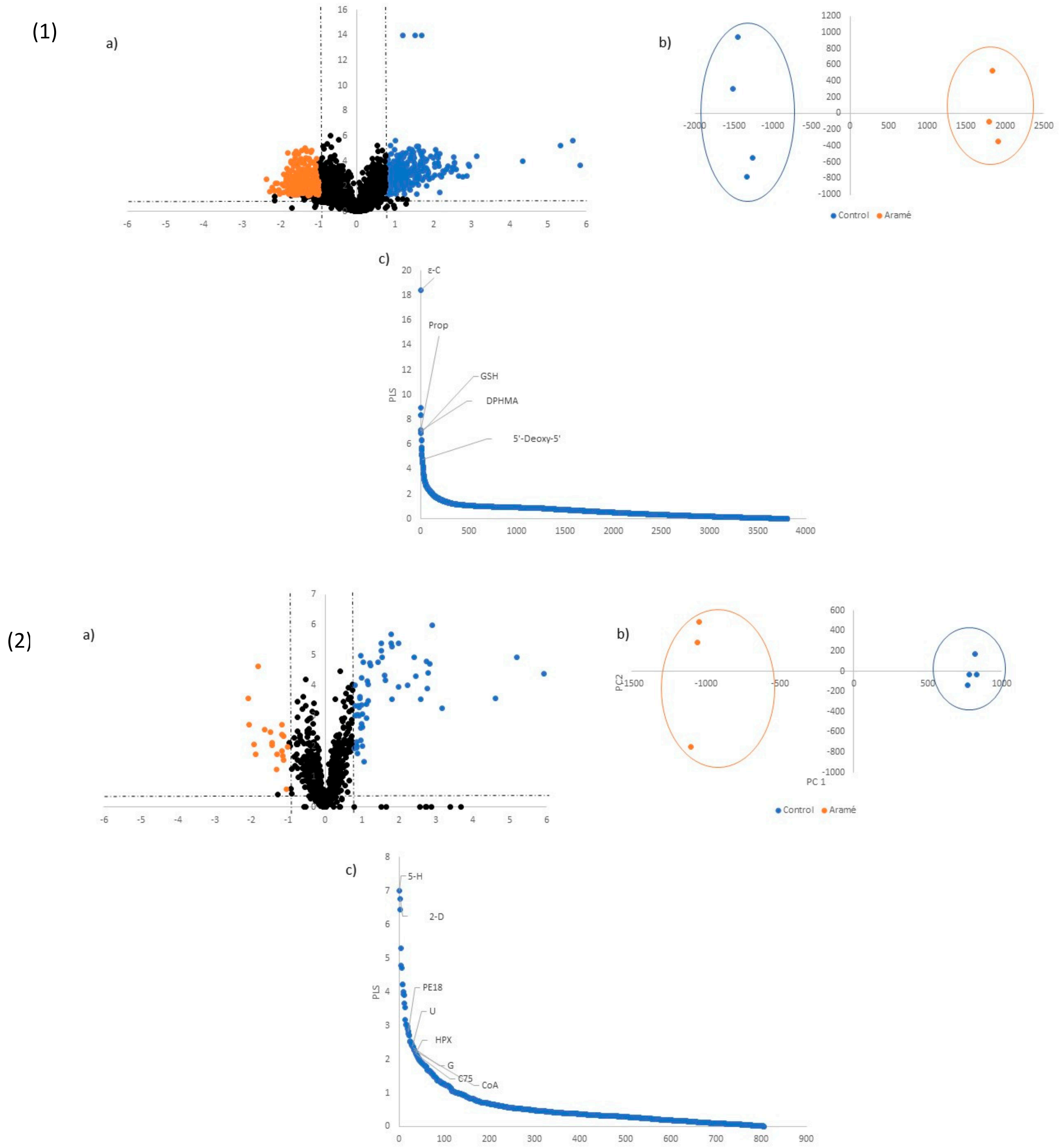

Figure S2. Analysis of compounds that vary in the presence or absence of Aramé, for Hep-G2 cells, in positive (1) and negative (2) mode. (a) volcano plot; (b) PCA plot; (c) PLS plot. ε-C—ε-Caprolactam; HPX—Hypoxanthine; Prop—3-[7-[4-(2-acetamido-4-methyl-thiazol-5-yl)sulfonylpiperazino]-5-methyl-[1,2,4]triazolo[1,5-a]pyrimidin-6-yl]ethyl ester propionic acid; DPHMA—(1,4-dimethyl-4-propylheptyl)-(2-methylbutyl)amine; GSH—Glutathione; I—Inosine; 5′–Deoxy–5′—5'-methylthioadenosine; 5-H—5-hydroxyeicosatetraenoic acid; 2-D—2-Dodecylbenzenesulfonic acid; PE 18—1-(9Z-Octadecenoyl)-sn-glycero-3-Phosphoethanolamine; U—Uridine; G—1-(3-Ethylsulfinylcyclohexyl)-2-methyl-3-[2-[(5-methyl-2-thienyl)sulfonylamino]ethyl]guanidine; C75—C75 trans; CoA—coenzyme A.

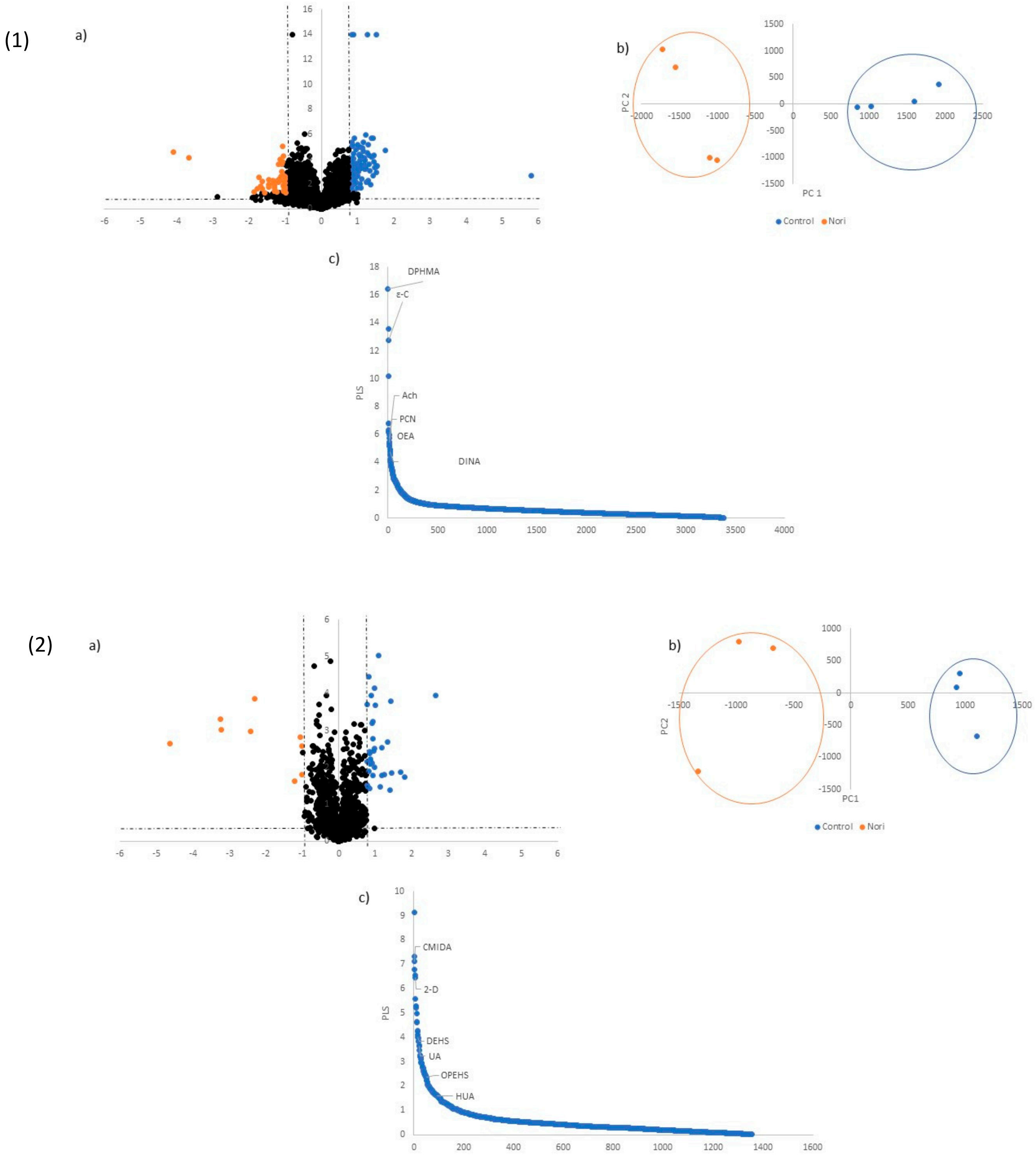

Figure S3. Analysis of compounds that vary in the presence or absence of Nori, for Caco-2 cells, in positive (1) and negative (2) mode. (a) volcano plot; (b) PCA plot; (c) PLS plot. DPHMA—(1,4-dimethyl-4-propylheptyl)-(2-methylbutyl) amine; ε-C—ε-Caprolactam; Ach—Acetylcholine; PCN—Phenacylamine; OEA—Oleamide; DINA—Dibenzyl-[4-(3,7-ditert-butyl-4H-diazepin-5-yl)phenyl]amine; CMIDA—2-keto-N-[6-(4-neopentylpiperazino)-3-pyridyl]-2-(2-phenyl-5,6,7,8-tetrahydroindolizin-3-yl)acetamide; 2-D—2-Dodecylbenzenesulfonic acid; DEHS—1-[1-(2-Dodekoxyethoxy)ethoxy]ethyl hydrogen sulfate; UA—Undecanedioic Acid; OPEHS—2-[3-[3-[3-(3-Octoxypropoxy)propoxy]propoxy]propoxy]ethyl hydrogen sulfate; HUA—2-hydroxy-10-undecanoic acid.

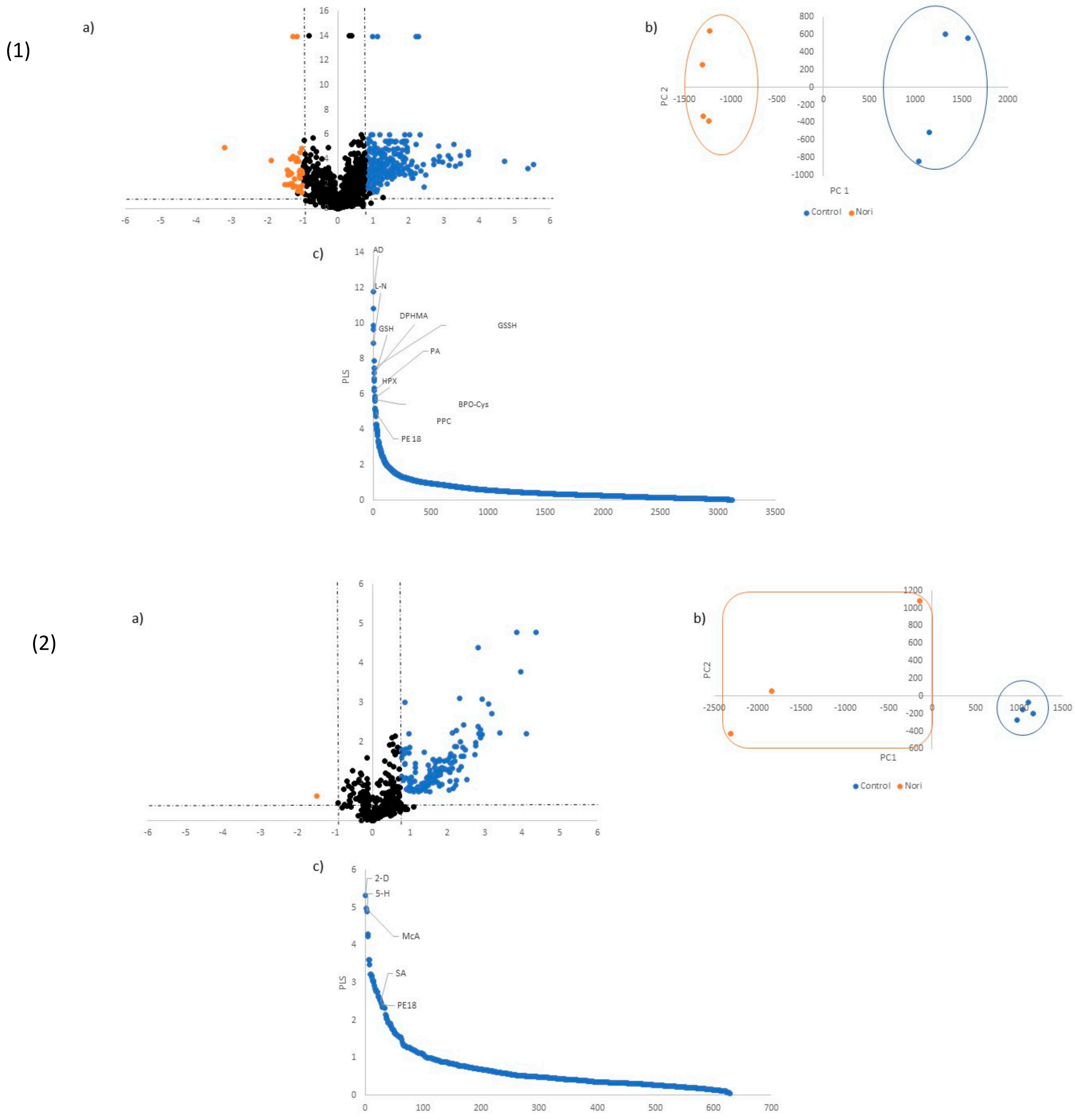

Figure S4. Analysis of compounds that vary in the presence or absence of Nori, for Hep-G2 cells, in positive (1) and negative (2) mode. (a) volcano plot; (b) PCA plot; (c) PLS plot. AD—Adenosine; L-N—L-Norleucine; GSSH—Glutathione; DPHMA—(1,4-dimethyl-4-propylheptyl)-(2-methylbutyl) amine; GSH—Glutathione; PA—Palmitic Amide; HPX—Hypoxanthine; BPO-Cys—Benzylpenicilloyl-Cysteine; PE18—1-(9Z-Octadecenoyl)-sn-glycero-3-Phosphoethanolamine; PPC—1-Palmitoylphosphatidylcholin; 2-D—2-Dodecylbenzenesulfonic acid; 5-H—5-hydroxyeicosatetraenoic acid; McA—Methoxyacetic Acid; SA—2-[[2-acetamido-3-carboxy-propanoyl]amino] succinic acid.
